# Ample Evidence: Dehydroepiandrosterone (DHEA) Conversion into Activated Steroid Hormones Occurs in Adrenal and Ovary in Female Rat

**DOI:** 10.1371/journal.pone.0124511

**Published:** 2015-05-11

**Authors:** Yingqiao Zhou, Jian Kang, Di Chen, Ningning Han, Haitian Ma

**Affiliations:** Key Laboratory of Animal Physiology and Biochemistry, College of Veterinary Medicine, Nanjing Agricultural University, Nanjing, China; Clermont Université, FRANCE

## Abstract

Dehydroepiandrosterone (DHEA) is important for human health, especially for women. All estrogens and practically half of androgens are synthesized from DHEA in peripheral tissues. However, the mechanism and exact target tissues of DHEA biotransformation in the female are not fully clear. The present study showed that maximal content of androstenedione (AD) and testosterone (T) were observed at 3h after DHEA administration in female rats, which was 264% and 8000% above the control, respectively. Estradiol (E2) content significantly increased at 6h after DHEA administration, which was 113% higher than that in control group. Gavage with DHEA could significantly reduce *3β-hydroxysteroid dehydrogenase (3β-HSD)* mRNA level at 3-12h and *17β-hydroxysteroid dehydrogenase (17β-HSD)* mRNA level at 12h in ovary, while increasing *aromatase* mRNA levels at 6, 24, and 48h. It is interesting that administration of DHEA caused a significant increase of *17β-HSD*, *3β-HSD* and *aromatase* mRNA levels in adrenal. The AD and T contents also markedly increased by 537% and 2737% after DHEA administration in ovariectomised rats, in company with a significant increase in *17β-HSD* and *3β-HSD* mRNA levels and decreased *aromatase* mRNA level in adrenal. However, DHEA administration did not restore the decreased E2, estrone (E1), and progesterone (P) caused by the removal of the ovaries in females. These results clearly illustrated that exogenous DHEA is preferentially converted into androgens in adrenal, while its conversion to estrogens mainly happens in the ovary through steroidogenic enzyme in female rats.

## Introduction

Dehydroepiandrosterone (DHEA) and its sulfaester (DHEA-S) are the most abundant circulating sex steroid hormones in women[1], mainly secreted by the adrenal glands, theca cells of ovarian follicle, and central nervous system[2]. In women, 75% of estrogen premenopausal and 100% of estrogens post-menopausal were transformed from DHEA [3]. As the only precursor of sex hormones for postmenopausal women, the levels of both DHEA and DHEA-S in serum decline with age. It is widely speculated that age-related decline of these C19 steroids might be caused by well-being, deterioration in cognition and lowered libido[4,5], or increased likelihood of low sexual function in both premenopausal and post-menopausal women[6]. It has been proposed that restoration of serum DHEA in women (60–79 years old) to the level found in young people may have anti-ageing effects[6]. Recently study indicated that DHEA supplementation could improve ovarian function, increases pregnancy chances and lowering miscarriage rates in women[7]. Now, DHEA is marketed as an important source of sex steroid in women and even in men[8].

A series of studies suggest that supplementation with DHEA in animal models and humans could have beneficial effects on multiple physiologic functions[9–13], resulting in widespread self-administration of DHEA in the USA as an anti-aging drugs, where it is considered to be a food supplement and is available without prescription. Previous studies mainly focused on the metabolism of exogenous DHEA in testis[14], Leydig cells[15], ovariectomy[16] or natural menopause[17]. However, little is known about the biological significance of dynamic biotransformation and trade-off relationship of exogenous DHEA among a large series of peripheral target tissues. How DHEA affects the contents of circulating steroid hormones and steroidogenesis-related enzymes expression levels in adrenal glands and ovary, and their potential relevance remain unclear.

As mentioned above, DHEA is administrated as a dietary supplement in clinical in the United and France, for its auxiliary effect on infertility in women and application value in premenopausal syndrome treatment[18]. However, it remains unclear about the biotransformation of DHEA in the body, and the research in this field will provide a theoretical basis for the clinical application of DHEA. Our previous study demonstrated that intraperitoneal injected DHEA (25mg·kg^-1^) in male rat over a 24-h time period could be converted into steroid hormones by steroidogenic enzymes[14]. Our present study was to investigate the amplitude and time course of major circulating active sex steroids and mRNA levels of steroidogenesis-related enzymes in adrenal glands and ovary, and their trade-off relationship in normal and ovariectomised female rats over 48 h period after DHEA(25mg·kg^-1^). This is necessitated to a deeper insight into the precise mechanisms of DHEA conversion into active androgens and estrogens in specific peripheral target tissues and by which exerts its biological actions in female animals.

## Materials and Methods

### Animals and experimental design

Two-month old female Sprague-Dawley (SD) rats weighing 200±20 g were purchased from Shanghai Experimental Animal Center of the Chinese Academy of Sciences (Shanghai, China). Two animals per cage were housed under constant temperature (25°C) and humidity (50%) and maintained on a 12-h light/dark cycle. Animals were maintained on standard rodent chow, and food and water were available *ad libitum*. All animal handling procedures were performed in strict accordance with guidelines established by Institutional Animal Care and Use Committee of Nanjing Agricultural University. Before initiation of experiment, rats were acclimatized to the environmental conditions for 1 week. A total of 70 female rats were randomly assigned to control group and DHEA-treated group. Rats were treated with DHEA (dissolved in DMSO) *via* gavage once at 25mg·kg^-1^ body weight and control group rats were received equal volume of vehicle. Rats were anaesthetized with ether and sacrificed at 0, 3, 6, 12, 24 and 48h, respectively. The ovary and adrenal glands were collected and stored at −80°C. Blood samples were taken and retained for subsequent analysis. An abbreviated experimental design is illustrated in Table 1.

**Table 1:** Abbreviated experimental design.^a^

Group	0 h	3 h	6 h	12 h	24 h	48 h
Control	n = 10	n = 6	n = 6	n = 6	n = 6	n = 6
DHEA(25mg/kg)		n = 6	n = 6	n = 6	n = 6	n = 6

^a^ 70 female rats were randomly assigned to 2 treatment groups and gavage with 0 (control), 25mg·kg^-1^ for 0,3,6,12,24,48h. At the conclusion of the experiment, rats were sacrificed and samples were taken and retained for subsequent analysis.

Another 30 female rats were randomly divided into three groups: Sham—operated (SO), Ovariectomised (OVX-control) and Ovariectomised+DHEA (OVX+DHEA). Rats were anaesthetized, and bilateral ovariectomy was performed for the OVX-groups through ventral approach. The fallopian tubes were tied up before the ovaries were removed. The sham-operated rats underwent the sham procedure, the ovaries were exposed and carefully manipulated, but they were left intact[19]. Ovariectomy was performed at least one week prior to the experiment. OVX-DHEA group rats were treated with DHEA *via* gavage once *at* 25mg·kg^-1^ body weight, SO and OVX-control groups rats received equal volume of vehicle. Rats were sacrificed at 6 h after gavage. The adrenal glands and blood samples were taken and retained for subsequent analysis.

### Serum steroid hormone measurements

Blood samples were allowed to clot at 4°C and centrifuged at 1520×g for 20min. The serum was harvested and stored at -20°C. Serum concentrations of testosterone (T), estradiol (E2), progesterone (P), cortisol (Cor) and aldosterone (Ald) were detected by using RIA kits according to the manufacturers’ protocol (Beifang Biotechnology Institution, China); Androstenedione (AD) and enstrone (E1) were detected by ChemiLuminescence (Beijing Biological Technology Institution, China). The antibody of T, E2, P, Cor and Ald is origin from guinea pig and there were no any immunological cross-reactivity between different hormones. The intra coefficients of variation for all hormone detection kit were less than 10% and the inter-assay coefficients of variation were less than 15%.

### Assay of steroidogenic enzymes expression by Real-time quantitative PCR

Total RNA was extracted from ovary and adrenal samples using Trizol reagent (TaKaRa, Japan) according to the manufacturer’s protocol and SYΒR Green was used as the dye in the Real-time quantitative. Reverse transcription was performed according to the method published previously [14]. In brief, an aliquot of cDNA sample was mixed with 25μL SYBR Green PCR Master Mix (TaKaRa, Japan), in the presence of 10pmol of each forward and reverse primers for β-actin (use as an internal control), *3β-hydroxysteroid dehydrogenase* (*3β-HSD*), *17β-hydroxysteroid dehydrogenase* (*17β-HSD*) and *aromatase* mRNA (Table 2), and then it was subjected to PCR under standard conditions (40 cycles). All samples were analyzed in duplicate using the ABI Prism 7300 Sequence Detection System (Applied Biosystems, Stockholm, Sweden) and programmed to conduct one cycle (95°C for 1min) and 40cycles (95°C for 30s, 60°C for 30s and 72°C for 40s). Fold change was calculated using the 2^-ΔΔCT^ method. The primes used were determined according to the published guideline[14] or designed by Primes Premier 5.

**Table 2:** Prime sequence of targeted gene and β-actin.

Gene	Primer sequences (5’–3’)	Orientation	Product size (bp)
β-actin	TCTGGCACCACACCTTCTA	Forward	186
	AGGCATACAGGGACAGCAC	Reverse	
17β-HSD	ATTGGAAGACTGACCGCCTACGAA	Forward	93
	CGCCGCTGTTTCCTCGATGCC	Reverse	
3β-HSD	ACCCTTTAACTGCCACTTGGTC	Forward	141
	AGTGTCCCGATCCACTCCGA	Reverse	
Aromatase	TTCTTACTCTCAGTTCGTGCTC	Forward	161
	GACCTATTTATTTGAAATGCACCA	Reverse	

### Data analysis and statistics

Data were analyzed with one-way ANOVA and expressed as mean values±SE. Treatment differences were subjected to a Duncan’s multiple comparison tests. Differences were considered significant at *P<0*.*05*. All statistical analyses were performed with SPSS 13.0 for Windows (StatSoft, Inc., Tulsa, OK, USA).

## Results

### Conversion of DHEA toward activated steroids hormone in normal female rats

**Fig 1:**
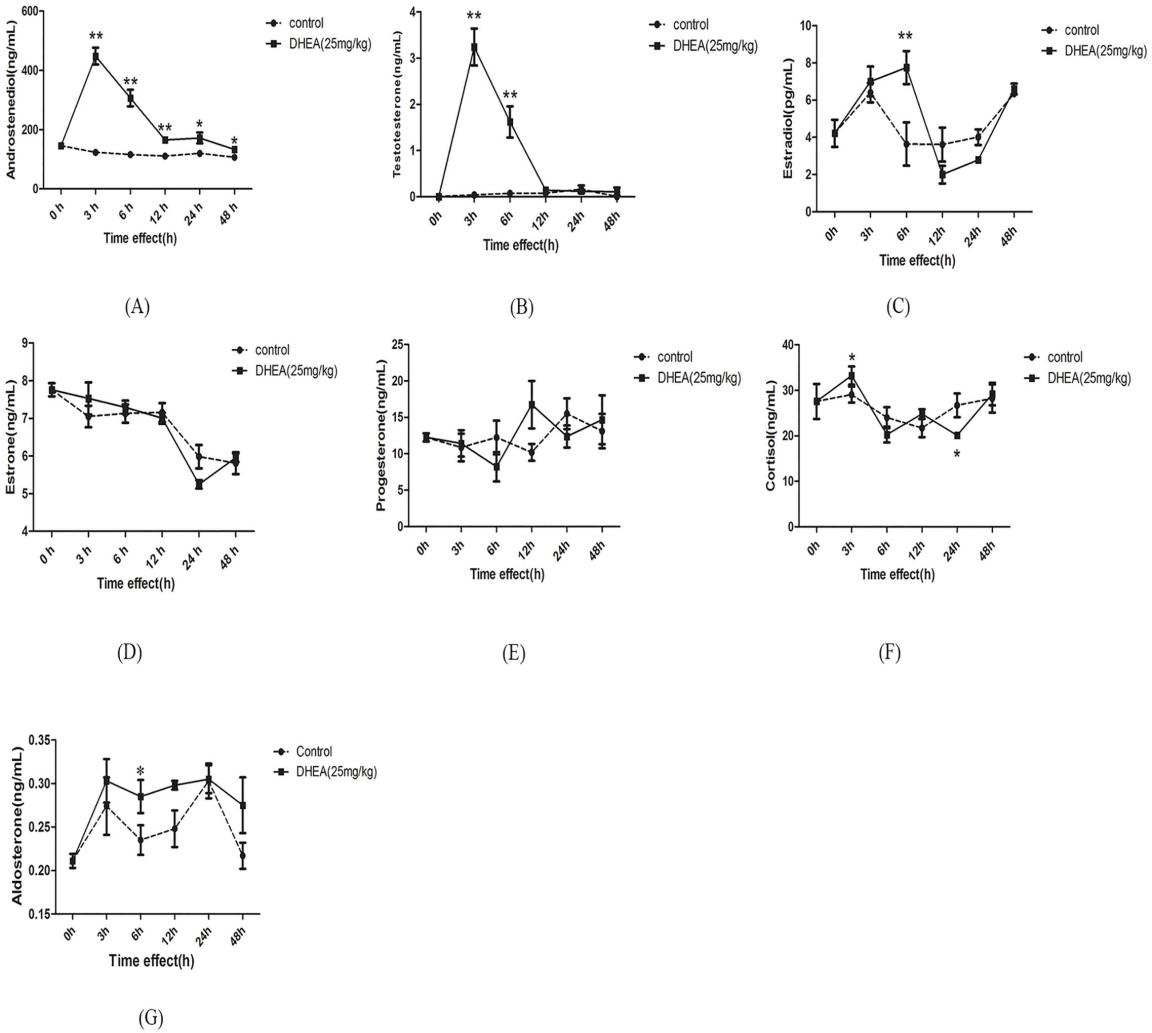
Serum contents of androstenedione (A), testosterone (B), estradiol (C), estrone (D), progesterone (E), cortisol (F) and aldosterone (G) in female rats following DHEA administration for 0h, 3h, 6h, 12h, 24h and 48h. Data are expressed as means±SE. (n = 6–10). **P < 0.*01* and *P < 0.*05*, compared with the respective control group at the same time interval.

As described in Fig 1, serum AD content markedly increased after DHEA administration over 3–48h period (*P<0*.*05*) and the maximal serum content of AD occurred at 3h (Fig 1A). T content markedly increased after DHEA administration over 3–6h period (*P<0*.*01*) and the maximal T content occurred at 3h (Fig 1B). Serum E2 content significantly increased at 6h after DHEA administration (*P<0*.*01*) than that control groups (Fig 1C). No significant differences were observed on the E1 and P contents over 3–48h period (Fig 1D and 1E). Serum Cor content significantly increased at 3h, but decreased again at 24h (*P<0*.*05*) (Fig 1F). Ald content significantly increased at 6h (*P<0*.*05*) after DHEA administration when compared to the control group (Fig 1G). These results indicated that exogenous DHEA is mostly converted into androgen in female rats.

### Assay of steroidogenic enzymes expression in normal female rats

**Fig 2:**
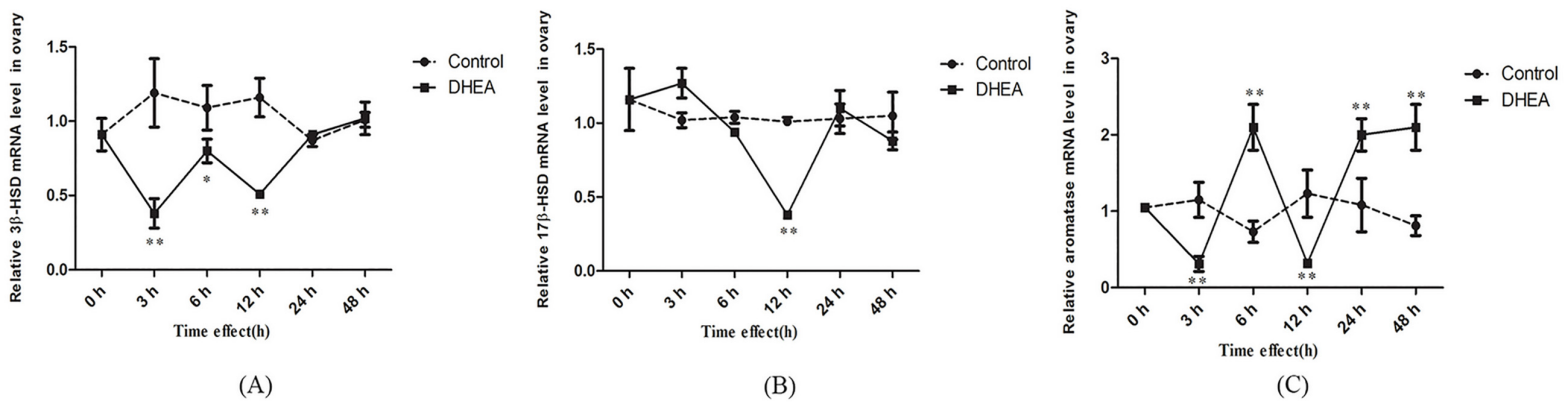
Effect of DHEA on *3β-HSD* (A), *17β-HSD* (B) and *aromatase* (C) mRNA levels in ovary in female rats. Data are expressed as means±SE. **P < 0.*01* and *P < 0.*05*, compared with the respective control group at the same time interval.

**Fig 3:**
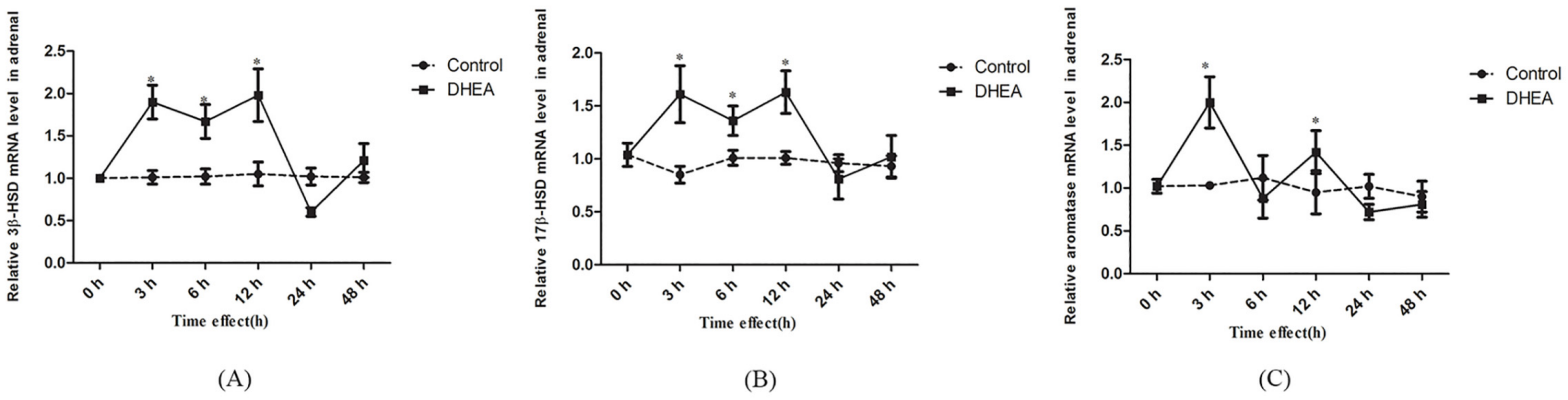
Effect of DHEA on *3β-HSD* (A), *17β-HSD* (B) and *aromatase* (C) mRNA levels in adrenal in female rat. Data are expressed as means±SE. **P < 0.*01* and *P < 0.*05*, compared with the respective control group at the same time interval.

As shown in Fig 2A, *3β-HSD* mRNA level in ovary significantly reduced *after DHEA administration* over 3–12h period (*P<0*.*05*). Compared with 3β-HSD, another steroidogenic enzyme, *17β-HSD* mRNA level only showed significant decreased at 12h after DHEA gavage (*P<0*.*01*) (Fig 2B). Although *aromatase* mRNA level also significantly decreased at 3h and 12h (*P*<0.01), a dramatic up-regulation occurred at 6h, 24h and 48h in ovary of female rats (*P<0*.*01*) (Fig 2C). It is interesting that oral administration of DHEA caused a significant increase of *17β-HSD*, *3β-HSD* and *aromatase* mRNA levels over 3–12h period in adrenal of normal female rats (*P<0*.*05*), no significant change of *aromatase* mRNA level was observed at 6h after DHEA administration (*P>0*.*05*) (Fig 3). The variation of mRNA levels of steroidogenic enzymes in ovary and adrenal might reflect the biotransformation dynamic and target tissues of exogenous DHEA.

### Conversion of DHEA toward androgen occurs in ovariectomised rats

**Fig 4:**
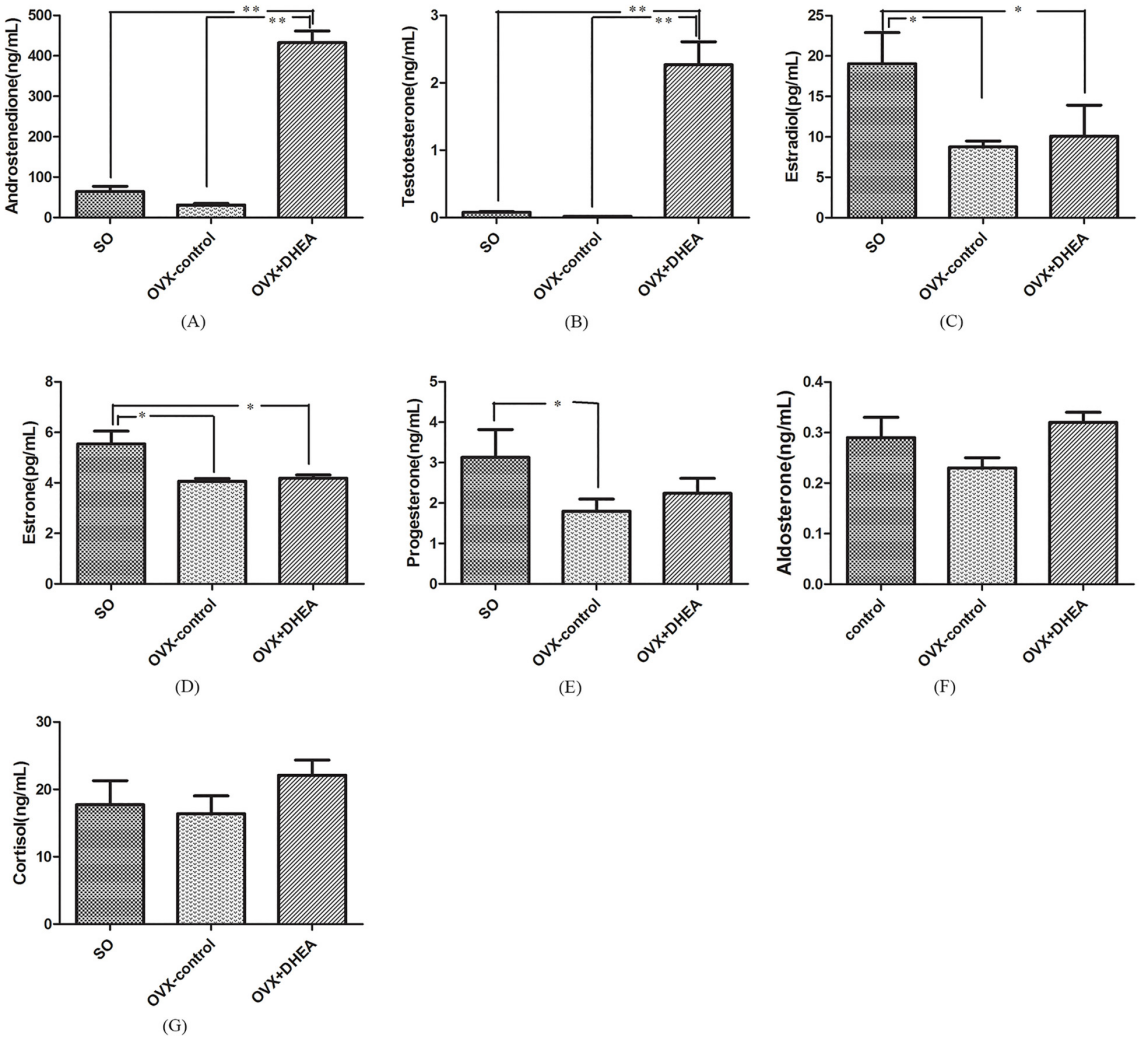
Serum contents of androstenedione (A), testosterone (B), estradiol (C), estrone (D), progesterone (E), aldosterone (F) and cortisol (G) in the ovariectomised (OVX) rats following DHEA administration for 6h. Data are expressed as means±SE. (n = 10). **P < 0.*01* and *P < 0.*05*, compared with the respective control group.

No significant changes were observed on the AD and T content in the OVX-control, compared with SO group (*P>0*.*05*). However, the AD and T contents markedly increased after DHEA administration when compared with the OVX-control (*P*<*0*.*01*) (Fig 4A and 4B). On the other hand, E2, E1 and P contents significantly decreased in the OVX-control (*P<0*.*05*), but gavage with DHEA did not restore the decrease of E2, E1 and P caused by the removal of the ovaries in female. (Fig 4C–4E). These results implied that DHEA conversion to androgen mainly occurs in the adrenal glands in female rats, while ovary is a critical target tissue for its conversion to estrogens.

### Assay of steroidogenic enzymes expression in adrenal in the ovariectomised rats

**Fig 5:**
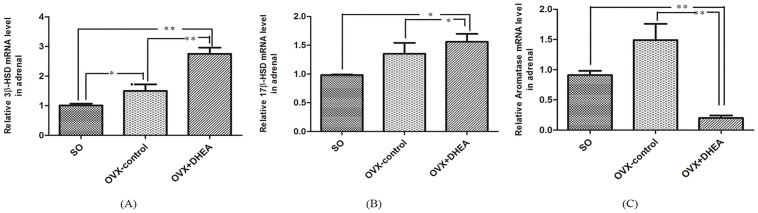
*3β-HSD* (A), *17β-HSD* (B) and *aromatase* (C) mRNA levels in adrenal in ovariectomised rats following DHEA administration for 6h. Data are expressed as means±SE. **P < 0.*01* and *P < 0.*05*, compared with the respective control group.

As shown in Fig 5, no significant differences were observed on *17β-HSD* and *aromatase* mRNA levels in the OVX-control compared with that in the SO group (Fig 5B and 5C), while there was a significant increase of *3β-HSD* mRNA level (*P<0*.*05*) (Fig 5A). Compared with OVX-control, *3β-HSD* (*P<0*.*01*) and *17β-HSD* (*P<0*.*05*) mRNA levels significantly elevated (Fig 5A and 5B), while *aromatase* mRNA level significantly decreased (*P<0*.*05*) after DHEA administration in ovariectomised rats (Fig 5C). These results indicated that DHEA could rapidly convert to AD and T via enhancing *17β-HSD*, *3β-HSD* and inhibiting *aromatase* expression in adrenal in ovariectomised rats.

## Discussion

**Fig 6:**
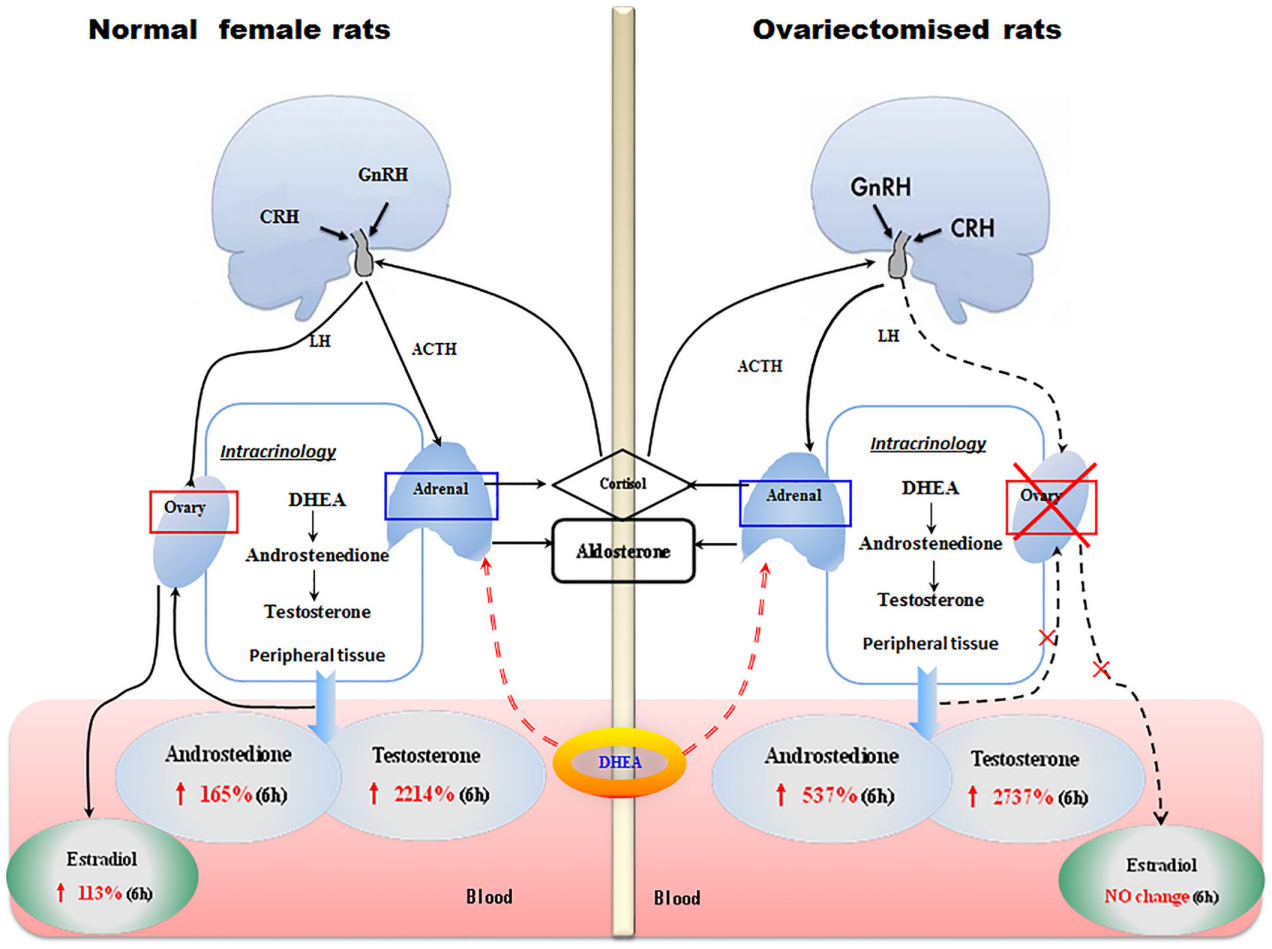
Conversion of exogenous DHEA in female rats. Left scheme represents the role of ovarian and adrenal on the exogenous DHEA conversion to activated steroid hormones in female rats. Serum androstenedione, testosterone and estradiol content significantly increased at 6h after DHEA administration. In adrenal glands, *3β-HSD* and *17β-HSD* mRNA levels were increased after DHEA administration, no difference change was observed on *aromatase* mRNA level. However, *3β-HSD* mRNA level were decreased and *aromatase* mRNA level increased in ovary after DHEA administration, there was no difference on *17β-HSD* mRNA expression levels. Right scheme represents the mainly peripheral target tissue of DHEA convert to activated steroid hormones in ovariectomised (OVX) rats, namely adrenal glands. Serum androstenedione and testosterone contents significantly increased in ovariectomised rats after DHEA administration, no changes were observed on estradiol and estrone content. The *3β-HSD* and *17β-HSD* mRNA levels were increased in adrenal after DHEA administration, while *aromatase* mRNA level was decreased.

Present study showed that serum AD and T contents significantly increased after DHEA administration in the female rats. These results were in agreement with the results of Panjari *et*.*al*[20], which suggest that the women randomly treated with 50mg oral DHEA daily for 4 months showed significant increases of serum AD and T content. Liu *et*.*al* [21] also confirmed this result. The maximal content of AD and T in the DHEA-treated group was increased by 264% and 8000% above control levels, and the maximal content of E2 also increased by 113% compared to control. The increase of serum androgens following DHEA administration is much larger than serum estrogen in female rats. These results indicated that DHEA gavage to female rats predominantly led to formation of androgen but not estrogen (Fig 6). It was previously reported that DHEA was preferentially metabolized to androgen in women and estrogen in men[8,17]. Present study also showed that, when compared to androgen, the appearance of maximal serum concentration for estradiol was delayed. DHEA could be rapidly converted into AD by *3β-HSD* in peripheral target tissues and then undergoes further conversion to T or E2 by *17β-HSD* and *aromatase*, respectively[3,17]. It is generally acknowledged that the expression level of *3β-HSD*, *17β-HSD*, *aromatase* and the hormone changes have potential correlation. A study showed that androgens up-regulate *aromatase* gene expression in purified adult rat germ cells whereas estrogens exert an opposite effect[22]. In our study, a rapid rise of AD and T in serum were in parallel with a significant increase in the *3β-HSD*, *17β-HSD* and *aromatase* mRNA levels in adrenal after DHEA administration, while *3β-HSD* and *aromatase* mRNA levels in ovarian decreased, we speculated that the down-regulation of aromatase mRNA level in ovarian may be due to the feedback of androgen in female rats. However, further study is needed to validate this hypothesis more precisely. Based on the levels of steroidogenic enzymes mRNA and hormone content changes, present results indicated that androgens production originates from the adrenal rather than ovary after treatment with DHEA in female rats.

To further confirm that androgen production was mainly originated from the adrenal, ovariectomised rats were used as a model to investigate the effect of DHEA administration on circulating androgen, estrogen and their conjugated metabolites. Since that most of serum steroid hormone contents had been affected at 6h after DHEA administration in female rats, serum steroid hormone contents and mRNA levels of adrenal steroidogenic enzymes were detected in ovariectomised rats at 6h after DHEA administration. The results showed that DHEA gavage caused a significant increase of serum AD and T contents than that of OVX-control (Fig 6). Similar result was also found in ovariectomized cynomolgus monkey 1h after DHEA administration [23]. DHEA gavage did not restore the decrease of E2, E1 and P contents due to the removal of the ovaries in female, while it could enhance the *3β-HSD* and *17β-HSD* mRNA levels and decreased the *aromatase* mRNA level in adrenal of ovariectomised rats. Taken together, ours results showed that biotransformation of exogenous DHEA to androgen mainly occurs in adrenal where it functions through upregulating the expression of *3β-HSD* and *17β-HSD* mRNAs.

In premenopausal women, >95% of serum E2 and most of serum E1 is derived from ovarian secretion, and E1 level generally parallel with those of E2. In the present study, the data suggested that DHEA administration could markedly increase serum E2 content in the normal female rats. It is interesting that serum E2 content was decreased by 46% in ovariectomised rats, while E2 content did not restore after DHEA administration. These results demonstrated that ovary plays a critical role in the transformation of DHEA to estrogen in female rats. Aromatase is important rate-limiting enzyme in the synthesis of E2, which is also a crucial steroidogenic enzyme in the irreversible conversion of androgens into estrogens[24,25]. Gonadotropin releasing hormone (GnRH) can stimulate the pituitary to produce follicle stimulating hormone (FSH) and luteinizing hormone (LH), thereby maintain the normal physiological function of ovarian by regulating various steroid hormone content; In turn, this two gonadotropins generate feedback information to the hypothalamus or pituitary. Present study showed that *aromatase* mRNA level has a low-high-low change in ovaries after gavage with DHEA, while its variation is just opposite in adrenal. These results indicated that conversion of DHEA to estrogen in ovary might be related to the bidirectional regulation of hypothalamus-pituitary-gonadal axis [26,27]. *Bourguiba*[22] reported *aromatase* gene expression was up-regulated by androgen in germ cells from adult rats, while estrogen exerts an opposite effect. In Leydig cells, addition of LH or T also induced a dose-related increase of *aromatase* transcription[28]. It is noteworthy that the increase of E2 content parallels with *aromatase* mRNA level in the ovary after DHEA administration in female rats, which is opposite in adrenal. No noticeable change in E2 content was found in ovariectomised rats after DHEA administration, and the *aromatase* level in adrenal was significantly decreased than OVX-control group. Taken together, the above data indicated that DHEA administration increases the mRNA level of *aromatase* in ovary, which subsequently accelerated the transformation of androgens to estrogens. Also, these results hinted that the conversion of exogenous DHEA depend on sex and target tissues, that the negative feedback effect of steroid concentrations on key enzymes in different tissues should also be considered.

In summary, our findings demonstrate that (a) exogenous DHEA in female rats is preferentially transformed into androgens, which mainly occurs in adrenal glands through increasing *3β-HSD*, *17β-HSD* expression and inhibiting their expression in ovary, and (b) the conversion of DHEA to estrogen mainly happened in ovary by increasing *aromatase* expression (Fig 6). These results demonstrated that replacement therapies used to achieve youthful hormonal levels by DHEA supplementation should not only take into the circulating levels of steroid, also considered the peripheral target tissues of DHEA biotransformation.
